# Spontaneous common bile duct perforation due to chronic pancreatitis, presenting as a huge cystic retroperitoneal mass: a case report

**DOI:** 10.4076/1757-1626-2-6273

**Published:** 2009-09-08

**Authors:** Necdet Fatih Yaşar, Bekir Yaşar, Mahmut Kebapçı

**Affiliations:** 1 Department of General Surgery, Eskisehir Osmangazi University Faculty of Medicine, Meselik Campus26480 EskisehirTurkey; 2 Department of Radiology, Eskisehir Osmangazi University Faculty of Medicine, Meselik Campus26480 EskisehirTurkey

## Abstract

Spontaneous perforation of the bile duct, is a disease in which spontaneous perforation occurs in the wall of the extrahepatic or intrahepatic duct without any traumatic or iatrogenic injury and more often described in neonates. In this report, we present a 38-year-old female patient who underwent surgery due to an intraabdominal cystic mass. The diagnosis of spontaneous rupture of the common bile duct and huge retroperitoneal biloma was made by intraoperative abdominal exploration. The biloma was drained, ruptured portion of the common bile duct was primarily repaired over a T-tube.

## Introduction

Most common etiologic factors of the common bile duct ruptures are common bile duct stones and/or cysts, blunt or penetrating abdominal traumas, hepatobilliary operations and instrumentations [1]. There are only few reports of spontaneous common bile duct rupture cases in literature and these cases are mostly infants or children due to congenital anomalies whereas it is exceedingly rare in adults [2-4]. There are also a few cases of spontaneous choledochus rupture described in pregnancy [5]. Presentation of such patients is either insidious or acute. In acute cases, fulminant bile peritonitis with pain, vomiting, fever and abdominal distension is observed whereas painless abdominal distension, increasing jaundice and clay coloured stools are the main symptoms of patients presenting insidously. Here, we report a 38 years old female patient, presented with a complex cystic retroperitoneal mass as result of spontaneous common bile duct rupture due to chronic pancreatitis and successfully treated with surgical intervention.

## Case presentation

**Figure 1.**
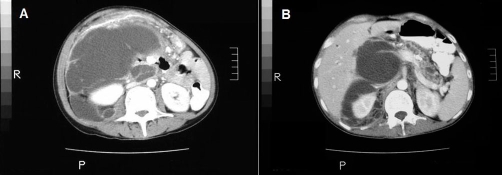
**(A and B)** Abdominal CT scan of the patient **(A, B)**: **(A)** Complex cystic retroperitoneal mass 20 cm in diameter, extending from liver to pelvis; **(B)** Dilatation of the intra and extrahepatic bile ducts, atrophy and parenchymal calsifications of the pancreas and dilatation of the Wirsung’s duct.

A 38-year-old, Turkish, Caucasian female patient presented with complain of intermittant cramping abdominal pain, nausea and vomiting which aggravated in the last 2 weeks. She had a history of cholecystectomy, performed 8 years ago. On physical examination, signs of anemia, jaundice and severe weight loss were inspected. The abdomen was distended and tender to palpation in the epigastrium and right upper quadrants with guarding, accompanied by reduced bowel sound. Laboratory examination revealed anemia (Hemoglobin: 8.1 g/dl), leukocytosis (13800/mm^3^), increase of erythrocyte sedimentation rate in first hour (105 mm/h), bilirubinemia (total bilirubin: 3 mg/dl and direct bilirubin: 1.82 mg/dl), increase of alchaline phosphatase (321 U/dl) and blood amilase (304 U/dl), prerenal azotemia (BUN: 50 mg/dl and creatinin: 1.24 mg/dl) and hypoalbuminemia (2.9 g/l). The levels of tumor markers were within the normal limits except CA-125, which was increased to 111 IU/dl. The abdominal ultrasound and computed tomography scan revealed a complex cystic retroperitoneal mass, 30 cm in diameter, extending from the subhepatic space down into the pelvis and was thought to be arised from the head of the pancreas. Dilatation of the intrahepatic and extrahepatic bile ducts and the Wirsung’s duct were accompanied by parenchymal calcifications and atrophy of the pancreas. The mass involved the porta hepatis and vascular structures, compressed the celiac trunk and superior mesenteric artery. The collecting system of the right kidney was dilated (Figure 1A and B).

**Figure 2.**
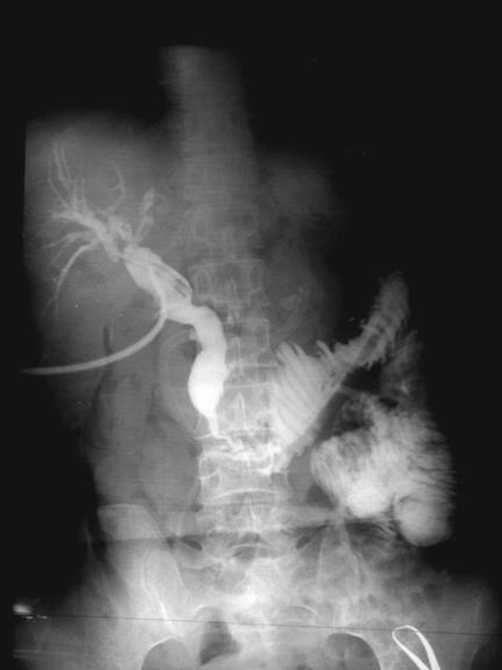
Peroperative Cholangiography of the patient: A stenosis of the distal common bile duct with well-arranged margins and regular thinning, revealing chronic pancreatitis.

After one week of supportive treatment and transfusion of 2 units of blood, the patient underwent surgery. The exploration of the abdomen revealed a cystic mass, extending from the liver to the pelvis retroperitoneally, which elevated the cecum, ascending colon and partially transverse colon. The mass was perforated during the dissection and an abundant bile drainage was observed. The frozen section of the biopsy, taken from the capsule of this cystic mass showed no evidence of malignancy. Thereafter, the mass was thought to be a huge retroperitoneal biloma. The porta hepatis, the common bile duct and the duodenum were explored. In the retropancreatic portion of the common bile duct, a perforation site with a dimension of 3 × 3 cm was inspected and a Foley catheter was placed through this hole for a peroperative cholangiography which revealed dilatation of intrahepatic bile ducts was and normal level of biliary tract drainage into the duodenum. Thereafter, the retroperitoneum was cleansed with saline and drained and biloma was excised. The perforation site was repaired with 3/0 silk sutures with omentoplasty. T-tube placement was performed on the proximal choledochus and a peroperative cholangiography was repeated, which demonstrated a stricture in the common bile duct but free passage of contrast media into the duodenum (Figure 2).

No complication was observed during early follow-up. On the postoperative 10^th^ day, the T-tube was withdrawn following a cholangiography which revealed a sufficient passage of contrast medium into the duodenum and no leakage out of the biliary tract. There was no abdominal pathology, except findings of chronic pancreatitis and dilatation of the common bile duct, at ultrasonography imaging performed 3 months after the operation.

## Discussion

The most common perforation site of biliary tract is the gallbladder, which is typically associated with cholecystitis and cholelithiasis [6]. Spontaneous perforation of extrahepatic bile duct is an extremely rare condition which more often seen in infants and children. Among children, it is mostly due to common bile duct cysts, delivery traumas and pancreaticobiliary junction anomalies [4,7,8]. In adults, common bile duct ruptures are extremely uncommon conditions. Generally the etiologic reasons in adults are bile duct stones, which may increase the common bile duct pressure [9]. Other ethiologic factors are biliary diverticulum [10] and acute pancreatitis [11]. In a retrospective study of 11 cases of nontraumatic perforation of the common bile duct, Kang et al. reported that the primary diseases were common bile duct stones in 7, intrahepatic bile duct stones in 2, choledochal cyst and phytobezoar, each, in one of the cases [12].

Our case was subjected to cholecystectomy 8 years ago and neither common nor intrahepatic bile duct stones were found during the operation by either inspection and radiographically. Also, no evidence of any choledochal cyst or phytobezoar was explored during the operation. The only pathological finding was the calcification in the pancreas due to chronic pancreatitis, seen in the abdominal computed tomographic scan and this might be the cause of the stricture of the distal biliary duct which was observed in the cholangiography. We assumed that the increased ductal pressure in the common bile duct and the excessive friability of an oedematous common bile duct during the acute exacerbation of chronic pancreatitis, together may have led to perforation.
